# Infrequent *RAS* mutation is not associated with specific histological phenotype in gliomas

**DOI:** 10.1186/s12885-021-08733-4

**Published:** 2021-09-15

**Authors:** Yasuhide Makino, Yoshiki Arakawa, Ema Yoshioka, Tomoko Shofuda, Sachiko Minamiguchi, Takeshi Kawauchi, Masahiro Tanji, Daisuke Kanematsu, Masahiro Nonaka, Yoshiko Okita, Yoshinori Kodama, Masayuki Mano, Takanori Hirose, Yohei Mineharu, Susumu Miyamoto, Yonehiro Kanemura

**Affiliations:** 1 Department of Neurosurgery, Kyoto University Graduate School of Medicine, Kyoto, Japan; 2 Department of Biomedical Research and Innovation, Institute for Clinical Research, National Hospital Organization Osaka National Hospital, Osaka, Japan; 3 Department of Diagnostic Pathology, Kyoto University Graduate School of Medicine, Kyoto, Japan; 4 Department of Neurosurgery, National Hospital Organization Osaka National Hospital, Osaka, Japan; 5 Department of Neurosurgery, Kansai Medical University, Osaka, Japan; 6 Department of Neurosurgery, Osaka International Cancer Institute, Osaka, Japan; 7 Department of Central Laboratory and Surgical Pathology, National Hospital Organization Osaka National Hospital, Osaka, Japan; 8 Division of Pathology Network, Kobe University Graduate School of Medicine, Kobe, Japan; 9 Department of Diagnostic Pathology, Hyogo Cancer Center, Hyogo, Japan

**Keywords:** *RAS mutation*, Glioma, Astrocytoma, Oligodendroglioma, Ganglioglioma, Histological phenotype

## Abstract

**Background:**

Mutations in driver genes such as *IDH* and *BRAF* have been identified in gliomas. Meanwhile, dysregulations in the p53, RB1, and MAPK and/or PI3K pathways are involved in the molecular pathogenesis of glioblastoma. *RAS* family genes activate MAPK through activation of RAF and PI3K to promote cell proliferation. *RAS* mutations are a well-known driver of mutation in many types of cancers, but knowledge of their significance for glioma is insufficient. The purpose of this study was to reveal the frequency and the clinical phenotype of RAS mutant in gliomas.

**Methods:**

This study analysed *RAS* mutations and their clinical significance in 242 gliomas that were stored as unfixed or cryopreserved specimens removed at Kyoto University and Osaka National Hospital between May 2006 and October 2017. The hot spots mutation of *IDH1/2*, *H3F3A*, *HIST1H3B*, and *TERT* promoter and exon 2 and exon 3 of *KRAS*, *HRAS*, and *NRAS* were analysed with Sanger sequencing method, and 1p/19q codeletion was analysed with multiplex ligation-dependent probe amplification. DNA methylation array was performed in some RAS mutant tumours to improve accuracy of diagnosis.

**Results:**

*RAS* mutations were identified in four gliomas with three *KRAS* mutations and one *NRAS* mutation in one anaplastic oligodendroglioma, two anaplastic astrocytomas (*IDH* wild-type in each), and one ganglioglioma. *RAS*-mutant gliomas were identified with various types of glioma histology.

**Conclusion:**

*RAS* mutation appears infrequent, and it is not associated with any specific histological phenotype of glioma.

**Supplementary Information:**

The online version contains supplementary material available at 10.1186/s12885-021-08733-4.

## Background

Glioma is a common tumour originating in brain [1]. Glioblastoma is the most aggressive subtype and the most common in adult glioma [1]. Other than glioblastoma, diffuse gliomas include astrocytomas and oligodendrogliomas. and anaplastic astrocytomas and anaplastic oligodendrogliomas show poor prognosis compared in each subtype [1]. These subtypes had been classified mainly by histological diagnosis [2]. Recent intensive genomic and molecular biological analyses of gliomas have identified several significant driver gene mutations in *IDH*, *BRAF*, or *H3F3* [3, 4]. Dysregulations in the p53, RB1, and MAPK / PI3K pathways have also been suggested to be involved in the molecular pathogenesis of glioblastoma [5, 6]. The importance of the molecular information to an understand the biological properties and pathogenesis of glioma is well recognized. The new 2016 World Health Organization (WHO) classification for central nervous system tumours has introduced the concept of multi-layered integrated diagnosis using a combination of traditional histopathological classification and information obtained from modern molecular analytical methods; therefore, the necessity for molecular information will increase in the neuro-oncological field [7].

*RAS* genes including *KRAS*, *HRAS*, and *NRAS* are well-known oncogenic genes, and are involved in the ERK pathway, a subgroup of the MAPK pathway. Ligand-mediated activation of receptor tyrosine kinases, such as epidermal growth factor receptor (EGFR), activate RAS proteins and initiate the cascade of the ERK signalling pathway. Activated RAS proteins activate the RAF, which can activate MEK just upstream of ERK [8, 9]. In addition, *RAS* genes also activate PI3K [10]. Through these several pathways, *RAS* genes promote cell proliferation, survival, and growth.

Mutations in *RAS* genes have been found in various cancer cells and lead to dysregulation of cell proliferation to promote oncogenesis [11, 12]. RAS proteins are bound to GDP in a stable state, and switch to an activated state when bound to GTP [12, 13]. GTPase switches GTP-bound RAS back to GDP-bound RAS [13]. *RAS* mutations have an impaired intrinsic GTPase and are insensitive to GTPase-activating proteins; therefore, inhibiting the conversion of GTP to GDP resulting in dysregulated cell proliferation and oncogenesis [11–13]. *RAS* mutations are mainly observed in codons 12, 13 and 61, and often in pancreatic, colorectal, lung and thyroid cancers [14, 15]. *KRAS*-activating mutations are widely effective as predictors of resistance to anti-EGFR monoclonal antibodies in colorectal and lung cancer patients [15–18]. Anti-KRAS drugs have been under development [19, 20], and some clinical trials are ongoing [21]. *RAS* mutation is now an important biomarker and therapeutic target in these solid cancers.

In terms of central nervous system diseases, a recent study showed an important relationship between *RAS* mutations and cerebral arterio-venous malformations as a non-neoplastic pathology [22]. Although several reports have found a small number of cases bearing *RAS* mutations in various gliomas, the clinicopathological properties of these mutations have not been fully addressed [23–26]. This study analysed *RAS* mutations and their clinical significance in gliomas.

## Methods

### Patients and samples

Inclusion criteria for the present study were the local initial diagnosis of gliomas according to the 2007 WHO classification of central nervous system tumours, and frozen or fresh tumour tissues available for genetic analysis. The exclusion criteria were insufficient quality of results of genetic analysis, or clinical data, but no case was excluded. A total of 242 cases were enrolled, including 167 tumours operated on from July 2008 to October 2017 in Kyoto University Hospital, and 75 tumours operated on from May 2006 to March 2017 in Osaka National Hospital. Clinical data collected from each institution included age, sex, tumour location, extent of resection, clinical course including treatment protocol and dates of surgery, recurrence or progression, and death. Ki-67 index were analysed in 167 tumours which was operated in Kyoto University Hospital.

### Sanger sequencing

Tumour DNA was extracted from tumour specimens with NucleoSpin® Tissue (MACHEREY-NAGEL, Düren, Germany). Regions of interest for driver genes [23, 27–30] were amplified by PCR with gene-specific primers (Supplementary Table 1) and TaKaRa Ex Taq® (TAKARA BIO, Shiga, Japan) (*IDH1/2*, *H3F3A*, and *HIST1H3B*) or AmpliTaq Gold 360 (Thermo Fisher Scientific, Waltham, MA) (*TERT*p, *KRAS*, *HRAS*, and *NRAS*) using Applied Biosystems GeneAmp PCR System 9700 (Thermo Fisher Scientific). PCR products were purified by ExoSAP-IT (Affymetrix, Santa Clara, CA), then sequenced with sequencing primer (*IDH1*) or PCR forward primer as a sequencing primer (*IDH2*, *H3F3A*, *HIST1H3B*, *TERT*p, and exons 2 and 3 of *KRAS*, *HRAS*, and *NRAS*) and BigDye® Terminator V1.1 Cycle Sequencing Kit (Thermo Fisher Scientific) using the ABI 3130xL Genetic Analyzer (Thermo Fisher Scientific).

### MGMT promoter methylation analysis

*O6-methylguanine-DNA methyltransferase* (*MGMT*) promoter methylation was assessed by quantitative methylation-specific PCR (qMSP), in accordance with previous reports [31, 32]. Genomic DNA samples were processed using the EZ DNA Methylation Gold Kit (Zymo Research Corporation, Irvine, CA). The methylation status of samples was analysed by qMSP using the QuantStudio 12 K Flex Real-Time PCR System (Thermo Fisher Scientific) with POWER SYBR® Green PCR Master Mix (Thermo Fisher Scientific) and specific primers (Supplementary Table 1) [33] by the standard curve method. The cut-off for determining a hypermethylated state was set as > 1% [32].

### 1p/19q co-deletion

1p/19q copy number analyses were performed with multiplex ligation-dependent probe amplification (MLPA) according to the instructions from the manufacturer (SALSA MLPA KIT probemix P088; MRC-Holland, Amsterdam, the Netherlands [32, 34]. Raw data were analysed by Coffalyser.NET software (MLC-Holland).

### Integrated diagnosis

Using all molecular pathological information, all cases received integrated diagnoses according to the 2016 WHO classification for central nervous system tumours.

### DNA methylation array

DNA methylation profiles were examined by Filgen, Inc. (Aichi, Japan) using the Infinium® MethylationEPIC BeadChip system (illumina, San Diego, CA). Raw methylation data (idat files) were uploaded onto the MolecularNeuropathology.org website and compared to a reference cohort to then be classified into subgroups of the highest calibrated score for each sample [35].

### Statistical analysis

All statistical analyses were performed using JMP version 15 software (SAS institute INC). The continuous variates were analyses by Student’s t-test. For survival analysis, overall survival (OS) was defined as the interval between the initial operative day and the date of death or last follow-up date on which the patient was known to be alive. Survival data were analysed using the log-rank test and Cox regression analyses. Differences were considered significant for values of *p* < 0.05.

## Results

All 242 cases were classified by the 2016 WHO classification, and mutation status is shown in Table 1. The average age of all patients was 51.3 years (range, 4–85 years; standard deviation, 19.2 years), with 153 men and 89 women. *RAS* mutations were detected in four gliomas (1.65% of total cases).

**Table 1:** Number of mutations in each type of glioma

	Number of cases	Driver gene mutations					1p/19q codeleted	***MGMT***p hypermethyl	***RAS*** mutations		
		***IDH1***	***IDH2***	***H3F3A***	***HIST1H3B***	***TERT***p			***KRAS***	***HRAS***	***NRAS***
**DA,*IDH*-mutant**	**20**	**20**	**0**	**0**	**0**	**1**	**0**	**13**	**0**	**0**	**0**
**DA,*IDH*-wild type**	**18**	**0**	**0**	**0**	**0**	**8**	**0**	**7**	**0**	**0**	**0**
**AA,*IDH*-mutant**	**11**	**10**	**1**	**0**	**0**	**0**	**0**	**5**	**0**	**0**	**0**
**AA,*IDH*-wild type**	**32**	**0**	**0**	**0**	**0**	**15**	**0**	**10**	**1**	**0**	**1**
**GBM,*IDH*-mutant**	**4**	**4**	**0**	**0**	**0**	**0**	**0**	**2**	**0**	**0**	**0**
**GBM,*IDH*-wildtype**	**94**	**0**	**0**	**0**	**0**	**39**	**0**	**31**	**0**	**0**	**0**
**gliosarcoma**	**1**	**0**	**0**	**0**	**0**	**0**	**0**	**0**	**0**	**0**	**0**
**OD,*IDH*-mutant and 1p/19q codeleted**	**22**	**18**	**4**	**0**	**0**	**20**	**22**	**20**	**0**	**0**	**0**
**OD, NOS**	**4**	**2**	**1**	**0**	**0**	**3**	**0**	**2**	**0**	**0**	**0**
**AO,*IDH*mutant and 1p/19q codeleted**	**15**	**12**	**3**	**0**	**0**	**15**	**15**	**12**	**1**	**0**	**0**
**diffuse midline glioma,*H3K27M*mutant**	**9**	**0**	**0**	**8**	**1**	**1**	**0**	**1**	**0**	**0**	**0**
**ganglioglioma**	**1**	**0**	**0**	**0**	**0**	**0**	**0**	**0**	**1**	**0**	**0**
**PA**	**7**	**0**	**0**	**0**	**0**	**0**	**0**	**1**	**0**	**0**	**0**
**PMA**	**1**	**0**	**0**	**0**	**0**	**0**	**0**	**0**	**0**	**0**	**0**
**PXA**	**2**	**0**	**0**	**0**	**0**	**0**	**0**	**0**	**0**	**0**	**0**
**APXA**	**1**	**0**	**0**	**0**	**0**	**0**	**0**	**0**	**0**	**0**	**0**
**Total**	**242**	**66**	**9**	**8**	**1**	**102**	**37**	**104**	**3**	**0**	**1**

*DA* Diffuse astrocytoma, *AA* Anaplastic astrocytoma, *GBM* Glioblastoma, *OD* Oligodendroglioma, *AO* Anaplastic oligodendroglioma, *PA* Pilocytic astrocytoma, *PMA* Pilomyxoid astrocytoma, *PXA* Pleomorphic xanthoastrocytoma, *APXA* Anaplastic pleomorphic xanthoastrocytoma, *TERT*p *TERT* promoter, *MGMT*p *MGMT* promoter

Mutations in *KRAS* were revealed in three tumours: an anaplastic astrocytoma, *IDH-*wildtype; an anaplastic oligodendroglioma, *IDH*-mutant with a 1p/19q codeletion; and a ganglioglioma. Another anaplastic astrocytoma, *IDH-*wildtype, showed *NRAS* mutation. No *HRAS* mutations were found in the present study. The clinical courses of four cases with RAS mutation are presented below, and summarized in Table 2. All four gliomas occurred in patients under 55 years old (average age, 41.5 years; range, 31–54 years) and were in the supratentorial area. *RAS*-mutant gliomas accounted for 6.25% of cases of anaplastic astrocytoma, *IDH*-wildtype (2 of 32), 6.67% of anaplastic oligodendroglioma, *IDH*-mutant with 1p/19q codeletion (1 of 15), and all gangliogliomas (1 of 1). Ki-67 labelling index of 3 WHO grade III tumours with *RAS* mutation was higher than that of other grade III tumours (average 29.2% (12.5–40%) vs 15.7% (3.6–50%), *p* = 0.04), and that of 2 anaplastic astrocytomas was higher than that of other anaplastic astrocytomas (average 37.5% (35–40%) vs 13.9% (3.6–30%), *p* = 0.0003). Ki-67 labelling index (5%) of the one ganglioglioma with *RAS* mutation was similar to that (average 3.38% (0.4–10%)) of other WHO grade I tumours.

**Table 2:** Summary of the four cases of *RAS*-mutant glioma

	Age	Sex	Location	Diagnosis	***RAS*** mutation	Other genetic profile	PFS (months)	OS (months)
**Case 1**	**31**	**F**	**frontal**	**AO-*IDH*mut + 1p/19qcodel**	***KRAS*G12A**	***IDH1*R132H,*TERT*C250T, 1p/19q codeletion**	**45**	**69+**
**Case 2**	**54**	**F**	**frontal, genu of corpus callosum**	**AA-*IDH*wild type**	***KRAS*E76D**	***MGMT*promoter hyper methylation**	**29**	**49**
**Case 3**	**45**	**M**	**frontal, parietal**	**AA-*IDH*wild type**	***NRAS*Q61R**		**3**	**24**
**Case 4**	**36**	**M**	**occipital**	**Ganglioglioma**	***KRAS*Q61K**		**18**	**32+**

*AA* Anaplastic astrocytoma. *AO* Anaplastic oligodendroglioma, *PFS* Progressive free survival, *OS* Overall survival

The clinical courses for each case were not uncommon. But the meaning of *RAS* mutations in glioma for survival were difficult to be discussed in the present study due to the small number of patients, and the Kaplan-Meyer curve showed no difference in overall survival between anaplastic astrocytoma, *IDH*-wild type, with and without *RAS* mutation (Supplementary Fig. 1).

## Case presentations

### Case 1

**Fig. 1:**
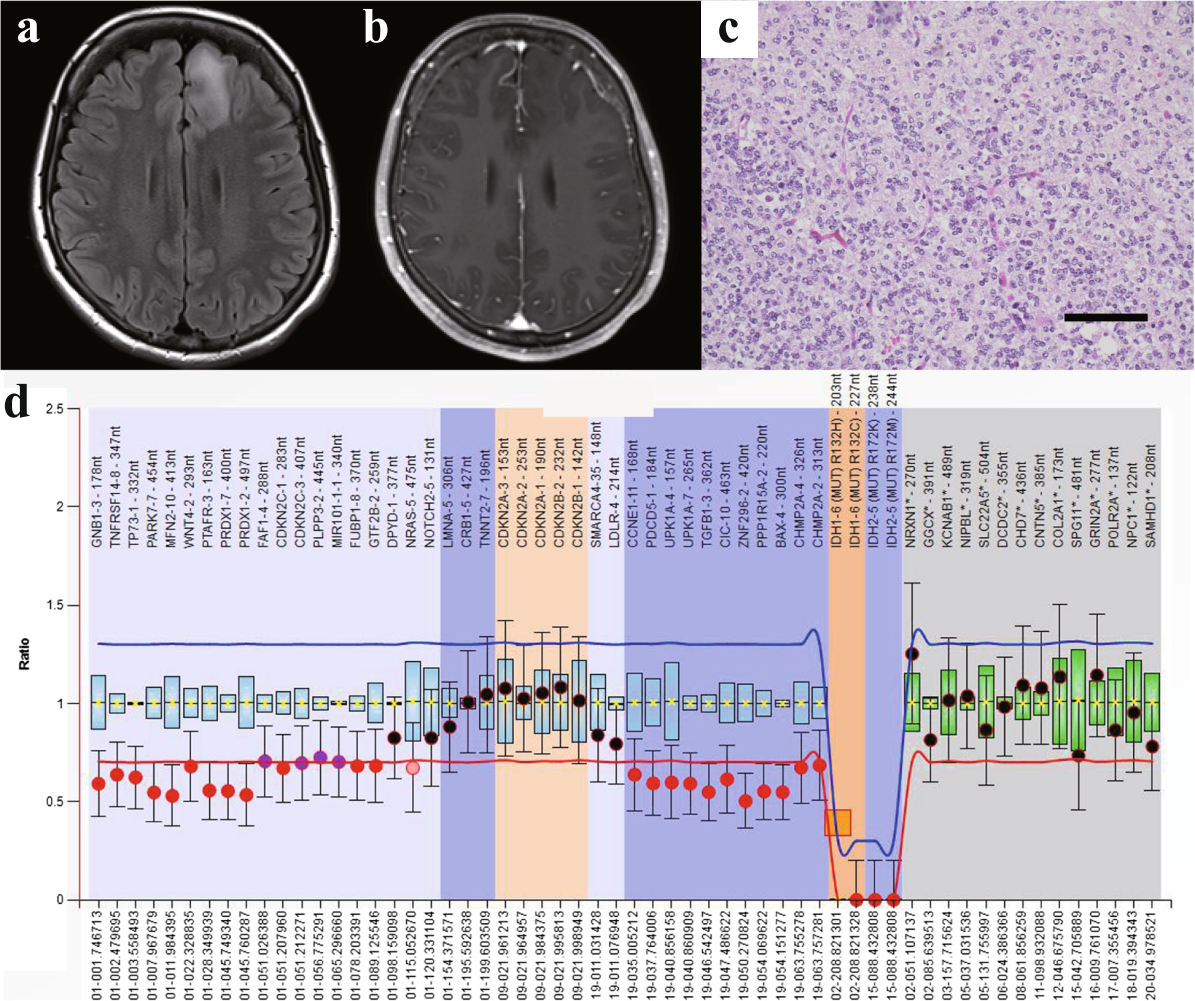
Neuroimaging, histopathological and molecular properties in Case 1. **a**, **b** Presurgical MRI for Case 1. A lesion is apparent in the left frontal lobe, with the appearance of widespread hyperintensity on fluid-attenuated inversion recovery (FLAIR) image (**a**) without any enhanced lesions on gadolinium-enhanced images (**b**). **c** Histopathological examination using hematoxylin and eosin staining. Findings typical of oligodendroglioma are observed. Some mitoses are evident. Scale bar, 100 μm. **d** MLPA analysis shows 1p/19q codeletion and no deletion of CDKN2A/B

A 26-year-old woman presented with a chief complaint of dizziness, and MRI showed left frontal lobe tumour with hyperintensity on T2-weighted imaging without gadolinium enhancement. She elected to follow a “wait and scan” approach (Fig. 1a, b). Five years later, the slowly growing tumour was removed under awake craniotomy. Post-operative MRI showed total resection of the T2-hyperintense lesion. Histopathological examinations detected atypical glia-like cells proliferating densely, cells with round nuclei and clear cytoplasm resembling fried eggs, as well as astrocytic cells, in a substantial area of the tumour. No necrosis or microvascular proliferation was identified (Fig. 1c). FISH detected 1p/19q codeletion, and Ki-67 labelling index of the tumour was 12.5%. The pathological diagnosis was anaplastic oligoastrocytoma, and the patient was followed without post-surgical chemotherapy or radiotherapy. At 45 months after the first surgery, the tumour recurred, and a second surgery was performed to achieve total resection. No re-recurrence was seen until this presentation, 69 months after the first surgery. No anti-tumour treatment had been performed after the second surgery. Genetic analysis of primary tumour showed *IDH1* R132H, *TERT* C250T, and *KRAS* G12A (Supplementary Fig. 2), and no mutations in *IDH2*, *H3F3A*, or *HIST1H3B*. *MGMT* promoter was hypomethylated. MLPA analysis showed 1p/19q codeletion and no *CDKN2A/B* deletion (Fig. 1d). The integrated diagnosis from Sanger sequencing, MLPA, and pathological findings was anaplastic oligodendroglioma, IDH-mutant and 1p/19q codeleted. Interestingly, genetic analysis of recurrent tumour showed the same result about *IDH1/2*, *TERT*p, *H3F3A* and *HIST1H3B*, but *KRAS* mutation was not detected.

### Case 2

**Fig. 2:**
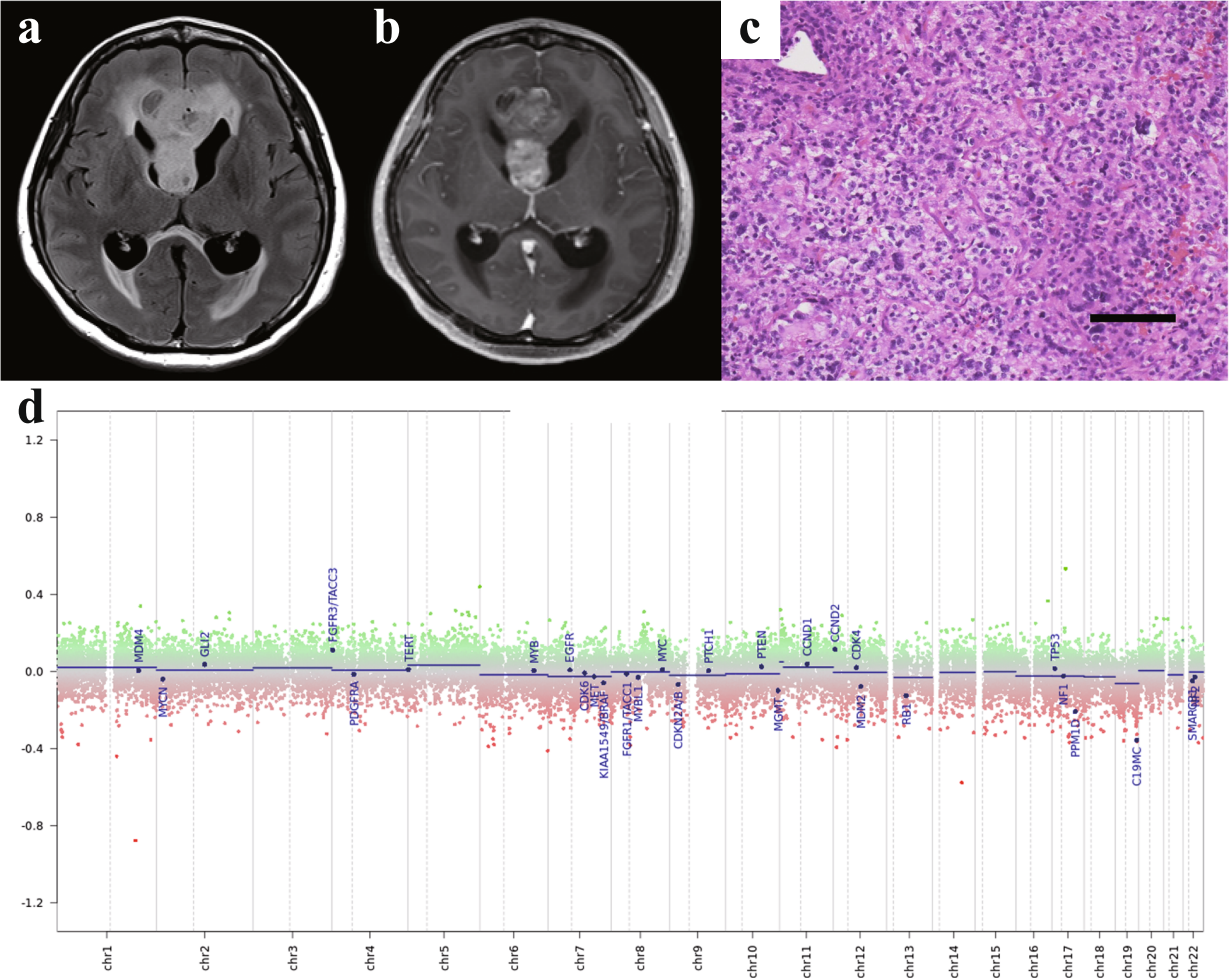
Neuroimaging, histopathological and molecular properties in Case 2. **a**, **b** Presurgical MRI for Case 2. Widespread hyperintensity is seen in bilateral frontal lobes and the corpus callosum to the occipital lobe on FLAIR image (**a**) with enhanced lesions in the genu of the corpus callosum and bilateral frontal lobes (**b**). **c** Histopathological examination using hematoxylin and eosin staining. Scale bar, 100 μm. **d** Copy number profile analysed by Illumina Methylation Epic Beadchip

A 54-year-old woman presented with a 3-month history of increasing headache and dizziness. MRI showed a gadolinium-enhanced lesion in the genu of the corpus callosum and a T2 hyperintensity lesion spreading to bilateral frontal lobes (Fig. 2a, b). Emergent endoscopic surgery was performed because of progressing hydrocephalus and achieved partial removal of the tumour. Histopathological examinations showed increased atypical glial cells and numerous mitoses, but no microvascular proliferation or palisading necrosis in the specimen (Fig. 2c). Ki-67 labelling index was 40%. The pathological diagnosis was high-grade glioma, and post-operative treatment was radiotherapy concomitant with temozolomide [36]. After discharge, she received maintenance therapy with temozolomide and bevacizumab. However, she showed progressive disease 29 months after the first surgery and received bevacizumab in combination with ifosfamide, carboplatin, and etoposide (ICE) [37]. The tumour kept growing slowly, and she died 49 months after the first surgery. Genetic analysis revealed no mutations in *IDH1/2*, *H3F3A*, *HIST1H3B* or *TERT* promoter, and *MGMT* promoter was hypermethylated. In addition, *KRAS* E76D was detected (Supplementary Fig. 2). A DNA methylation array showed *MGMT* promoter hypermethylation, matching the qMSP result, but did not identify any matching methylation classes with high calibrated scores. The copy number profile showed no special characteristics (Fig. 2d). The final diagnosis was anaplastic astrocytoma, *IDH*-wildtype. To support this diagnosis, additional Sanger sequencing was performed and *TP53* P72R was revealed.

### Case 3

**Fig. 3:**
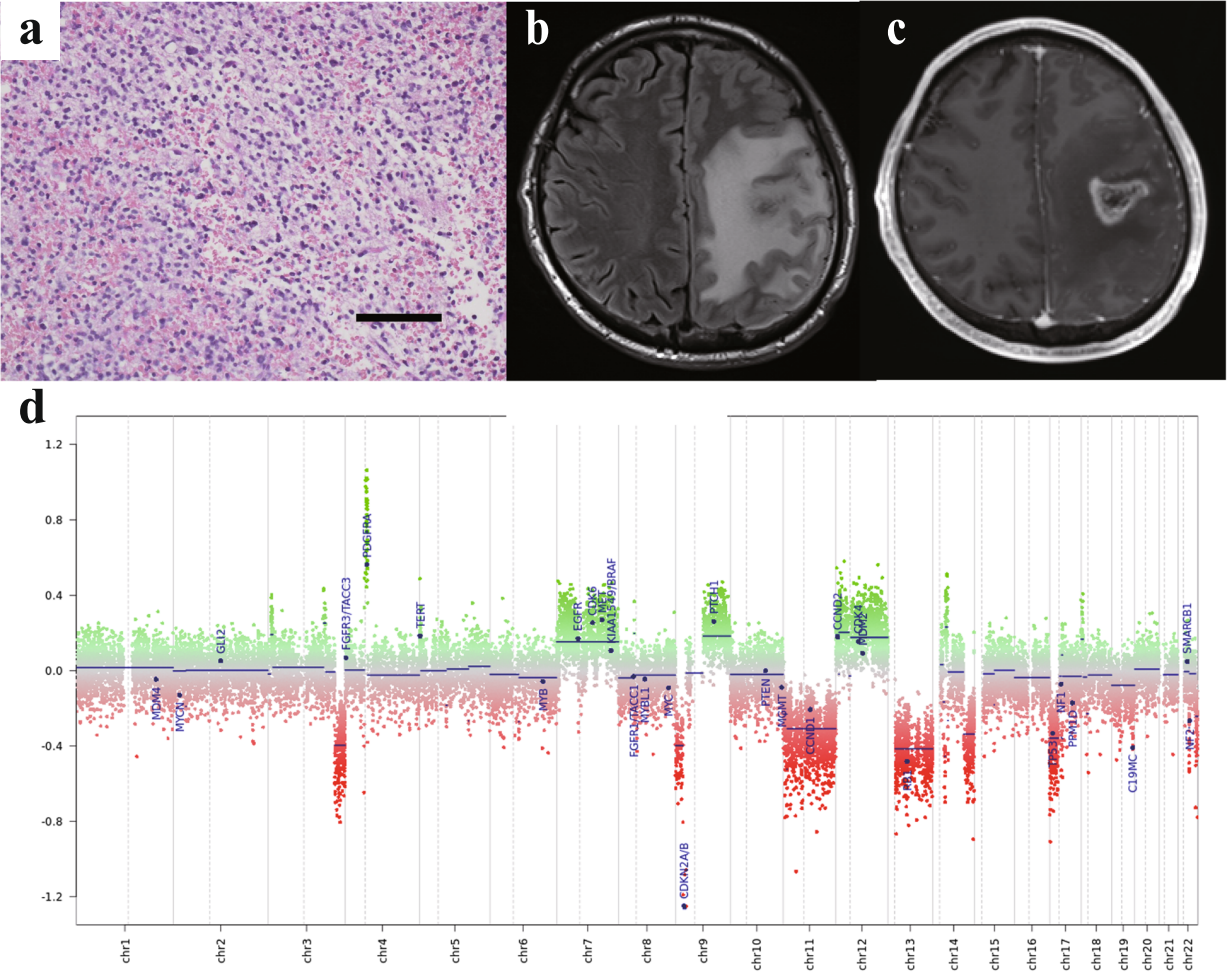
Neuroimaging, histopathological and molecular properties in Case 3. **a** Histopathological examination using haematoxylin and eosin staining. Scale bar, 100 μm. **b**, **c** MRI for Case 3 at the time of recurrence. Widespread hyperintensity is seen in the left frontal and parietal lobes on FLAIR imaging (**b**) with an enhanced region in the centre of the lesion (**c**). **d** Copy number profile as analysed by Illumina Methylation Epic Beadchip

A 45-year-old man presented with simple partial seizures involving the right side of the face. MRI showed a T2-hyperintense lesion without gadolinium enhancement in the left frontoparietal lobe. Histopathological examinations of stereotactic biopsy revealed tumour cells with semiround or round nuclei (Fig. 3a) of various sizes, and areas of mitoses, with a Ki-67 labelling index of 35%. No necrosis or vascular proliferation was seen, and FISH revealed no 1p/19q codeletion. The diagnosis was anaplastic glioma. He received chemoradiotherapy comprising 60 Gy with temozolomide, but MRI showed tumour progression 3 months later (Fig. 3b, c). He was treated with additional radiotherapy and bevacizumab with ICE but died 24 months after the first surgery. Genetic analysis revealed *NRAS* Q61R (Supplementary Fig. 2), but no mutations in *IDH1/2*, *H3F3A*, *HIST1H3B* or *TERT* promoter, and *MGMT* promoter was not hypermethylated. Methylation-based profiling by the DNA methylation array classified this tumour as “methylation class family Glioblastoma, *IDH* wildtype” with a calibrated score of 0.55. This low score could be a result of low tumour content or low DNA quality in the analysed material, but the classification matched well with the clinical course and pathological findings. The copy number profile showed amplification of *PDGFRA* and loss of *CDKN2A/B* and *TP53*, gain of chromosomes 7, 9q, and 12, and loss of chromosomes 11 and 13 (Fig. 3d). Because there was no evidence of grade 4 histology, the integrated diagnosis was determined as anaplastic astrocytoma, *IDH*-wildtype.

### Case 4

**Fig. 4:**
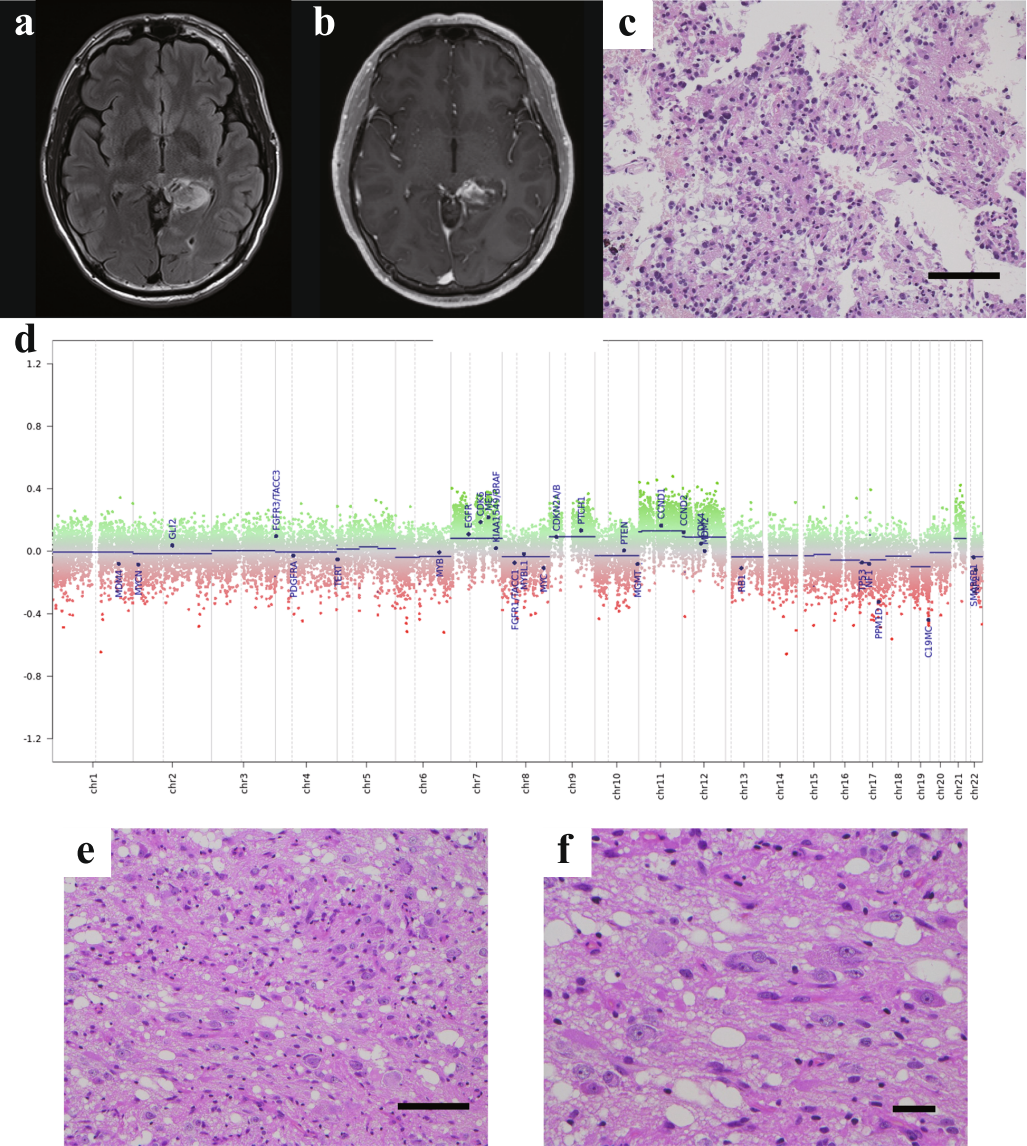
Neuroimaging, histopathology and molecular properties of Case 4. **a**, **b** Presurgical MRI for Case 4. FLAIR hyperintensity and gadolinium-enhanced lesions are seen in the left medial temporal to the occipital lobe. **c** Histopathological examination using haematoxylin and eosin staining. Scale bar, 100 μm. **d** Copy number profile as analysed by Illumina Methylation Epic Beadchip. **e**, **f** Histopathological examination using haematoxylin and eosin staining at the time of review. In this area, many large, ganglion-like cells are observed (**e**). Some cells show double nuclei (**f**) and some eosinophilic bodies are present. Scale bars, 100 μm (**e**), 20 μm (**f**)

A 36-year-old man was referred after a brain tumour was coincidentally identified on screening CT after a traffic accident. MRI revealed a left medial occipitotemporal tumour with gadolinium enhancement (Fig. 4a, b). Histopathological examination of stereotactic biopsy (Fig. 4c) revealed a dense, invasive proliferation of various-sized glial cells with some mitoses, and a Ki-67 labelling index of 5%. No necrosis or microvascular proliferation was identified. Immunohistochemistry showed positive results for olig2, GFAP, and p53, while FISH showed no 1p/19q codeletion. Based on these findings, the first diagnosis was anaplastic astrocytoma. The patient received chemoradiation and maintenance chemotherapy with temozolomide. As tumour progression was detected 18 months after biopsy, he underwent gross total resection of the tumour. No tumour recurrence was identified after the second surgery, and no additional treatment was performed for 24 months. Genetic analysis of primary tumour revealed *KRAS* Q61K (Supplementary Fig. 2), wild-type *IDH1/2*, *H3F3A*, *HIST1H3B* and *TERT* promoter, and no *MGMT* promoter hypermethylation. The DNA methylation array classified “methylation class family pilocytic astrocytoma” as the methylation class and “methylation class of low-grade glioma, subclass hemispheric pilocytic astrocytoma and ganglioglioma” as the methylation class family member, with calibrated scores of 0.97 and 0.96, respectively. The copy number profile showed gain of chromosomes 7, 9, 11 and 12 (Fig. 4d). Histopathological re-examination revealed many large ganglion cells with anisonucleosis and some double nuclei (Fig. 4e), Nissl bodies and eosinophilic granular bodies (Fig. 4f) in specimens from the second surgery. Given these genetic results and histopathological findings, the final diagnosis was ganglioglioma. Like as case 1, *KRAS* mutation was not detected in the recurrent tumour.

### Review of the previous reported cases

Previous 17 studies presented 44 gliomas with *RAS* mutations (Table 3). They were 17 glioblastomas (2 were glioblastomas with oligodendroglial component), 1 astrocytoma, 4 oligodendrogliomas, 3 anaplastic oligodendrogliomas, 1 oligoastrocytoma, 9 pilocytic astrocytomas, 2 anaplastic pilocytic astrocytomas, 2 fibrillary astrocytomas, 2 gangliogliomas, 2 pleomorphic xanthoastrocytomas, and 1 gliosarcoma. And they included 14 men and 13 women, and ages at diagnosis were described in 28 patients and they were 1–64 years (average, 33.3 years; standard deviation, 17.1 years). The co-existing mutations were various and *IDH1* R132H was the major mutation which detected in 11 cases.

**Table 3:** Review of the literature about *RAS*-mutant gliomas

Author (publish year)	Target of the study	Age	Sex	Histology	*RAS* mutation	Other mutations
Maltzman (1997) [38]	46 brain tumors	5	F	fibrillary astrocytoma	*NRAS* Q61E	
		12	F	fibrillary astrocytoma	*NRAS* Q61E	
		4	F	pilocytic astrocytoma	*NRAS* Q61E	
Knobbe (2004) [39]	94 glioblastomas	NA	NA	glioblastoma	*NRAS* G12D	
		NA	NA	gliosarcoma	*NRAS* G12D	
Janzarik (2007) [40]	25 astrocytoma	NA	NA	pilocytic astrocytoma	*KRAS* G12A	
Jeuken (2007) [41]	93 gliomas	NA	NA	anaplastic oligodendroglioma	*NRAS* G10E	
Schiffman(2010) [42]	41 astrocytomas	14	NA	glioblastoma	*KRAS* G12V	
Cin(2011) [43]	125 pilocytic astrocytomas	11	NA	Pilocytic astrocytoma	*KRAS* E63K	
		1	NA	Pilocytic astrocytoma	*KRAS* G12A	
Chi (2012) [24]	218 gliomas	NA	NA	glioblastoma	*KRAS* G61R	
		NA	NA	glioblastoma with oligodendroglioma component	*KRAS* G12A	*IDH1* R132H
		NA	NA	glioblastoma	*KRAS* G12C	
Theeler (2014 [44])	40 pilocytic astrocytoma	18	M	pilocytic astrocytoma	*NRAS* G12D	
		25	M	pilocytic astrocytoma	*NRAS* G12D	
		34	M	pilocytic astrocytoma	*NRAS* G12D	
		46	M	pilocytic astrocytoma	*KRAS* G12S	*BRAF*-*KIAA 1549* fusion
Milinkovic (2014 [45])	5 cerebellar glioblastomas	42	M	glioblastoma	*KRAS* c.7_8insT, *HRAS* T4H	*TP53* R273P
		41	M	glioblastoma	*KRAS* c.7_8insT, *HRAS* T4H	*TP53* exon9 deletion, G298D(second surgery)
		64	M	glioblastoma	*HRAS* T4H	
		36	M	glioblastoma	*KRAS* c.177_178insA, *HRAS* T4H	*TP53* C176R
		49	M	glioblastoma	*HRAS* exon2 insertion	
Wakimoto (2014) [26]	20 gliomas. IDH-mutant	60	M	oligoastrocytoma	*KRAS* G12R	*IDH1* R132H, *AKT1* E17K, *PIK3CA* H1047L, 1p/19q codeletion
		41	F	astrocytoma	*KRAS* G13D	*IDH1* R132H, 1p/19q codeletion
		33	F	oligodendroglioma	*KRAS* G13R	*IDH1* R132H, 1p/19q codeletion
		54	M	glioblastoma with oligodendroglioma component	*KRAS* G12A	*IDH1* R132H, 1p/19q codeletion
Samel (2016) [46]	case report	44	F	glioblastoma (high grade area)	*KRAS* exon2	*EGFR* exon18, *BRAF* exon15, *TP53*(IHC expression)
Ballester (2017) [47]	381 diffuse gliomas	NA	NA	anaplastic oligodendroglioma	*KRAS* G60S	*IDH1*R132H, *CDKN2A* P75L, *JAK3* G712D, *MPL* A519T
		NA	NA	anaplastic oligodendroglioma	*KRAS* G12D	
		NA	NA	glioblastoma	*KRAS* G12A	*IDH1* R132H, *TP53* Q104*
		NA	NA	glioblastoma	*KRAS* A146T	*IDH1* R132H, *CDKN2A* R58, *KIT* A814V, *PIK3CA* E545K, *TP53* G245S
		NA	NA	glioblastoma	*NRAS* Q61K	*TP53* E180K
		NA	NA	glioblastoma	*NRAS* Q61K	
		NA	NA	oligodendroglioma	*NRAS* G12C	*IDH1* R132H
Pekmezci (2018) [25]	40 gangliogliomas	32	F	ganglioglioma	*KRAS* Q61K	
		24	M	ganglioglioma	*KRAS* Q61K	
Reinhardt (2018 [48])	64 anaplastic pilocytic astrocytomas	NA	F	anaplastic pilocytic astrocytoma	*KRAS* Q61H	*MAPK* alteration, *CDKN2A/B* deletion/mutation, *ATRX* loss/mutation, *MGMT*p methylation
		NA	F	anaplastic pilocytic astrocytoma	*KRAS* V14A	*MAPK* alteration, *CDKN2A/B* deletion/mutation, *ATRX* loss/mutation, *MGMT*p methylation
Chau (2019) [49]	case report	24	F	pilocytic astrocytoma	*KRAS* E63K	*RAD1* F475L, *NOTCH3* S502F, *JAK1* c.6 + 1G > T
Shittenhelm (2019) [50]	186 gliomas	51	M	oligodendroglioma	*KRAS* G12R	*IDH1* mutant, 1p/19q codeletion, *TERT*p
		48	F	oligodendroglioma	*KRAS* G12C	*IDH1* mutant, 1p/19q codeletion, *TERT*p
		39	F	glioblastoma	*KRAS* Q61L	*TERT*p mutation
Zou (2019) [51]	13 pleomorphic xanthoastrocytomas	50	M	pleomorphic xanthoastrocytoma	*KRAS* Q61H	*NOTCH2* R2298W, *MEN1* G219, *CHEK1* P318A, *PARP4* L482F, *FANC1* L1253V, *FANCA* Q1437K, *TP53* R248Q, *NOTCH3* R1666W, *CCNE1* I298, *NOTCH1* N454
		29	F	pleomorphic xanthoastrocytoma	*KRAS* Q61K	

*NA* Not available

## Discussion

Various reports have described *RAS* mutations in glioma. Chi et al. analysed 214 gliomas, and they found 3 *KRAS* mutation cases among 164 glioblastomas [24]. Wakimoto et al. found 4 *KRAS*-mutant *IDH*-mutant gliomas, comprising 2 oligodendrogliomas, a grade 2 astrocytoma, and a glioblastoma with an oligodendroglial component. These 4 tumours all showed 1p/19q codeletions, and were thus considered to represent grade 2 or 3 oligodendrogliomas based on the 2016 WHO brain tumour classifications [26]. Ballester et al. showed the results of next-generation sequencing of 381 diffuse gliomas [47]. They found a *NRAS* mutation in 11 oligodendrogliomas, 2 *KRAS* mutations in 16 anaplastic oligodendrogliomas, and 2 *KRAS* mutations and 2 *NRAS* mutations in 226 glioblastomas. Pekmezci et al. detected *KRAS* mutation in 2 of 40 gangliogliomas [25]. Literature review of *RAS*-mutant gliomas showed that *RAS*-mutant gliomas have various histologies and that *RAS* mutation co-existed with other genetic alterations. They were often reported in young cases. The larger database made by the Cancer Genome Atlas (TCGA) Research Network showed 2 *KRAS* mutation and 2 *NRAS* mutation in 590 glioblastomas, and 1 *KRAS* mutation and 1 *NRAS* mutation in low grade gliomas with *IDH*-mutant and 1p/19q codeletion, and 1 *KRAS* mutation and 2 *NRAS* mutation in those with *IDH*-mutant and no 1p/19q codeletion [52]. Summarizing by age group, *RAS* mutations were found in 1 out of all 93 gliomas under 30 years old, 6 out of 631 cases from 30 to 60 years old, and 1 out of 363 cases in over 60 years old, and there was no significant difference in frequency of *RAS* mutations [52]. Similar to these studies, we report *RAS* mutation as a rare occurrence with no association to a particular histological phenotype of glioma. Additionally, copy number analysis in the present study revealed no chromosomal gain or loss.

In this study, *RAS*-mutant gliomas showed various histology, but all cases were in relatively young adults. *RAS* mutation was found in an anaplastic oligodendroglioma, two *IDH*-wildtype anaplastic astrocytomas, and a ganglioglioma. Among the 20- to 60-year-old patients of our present cohort, 14 tumours were anaplastic oligodendrogliomas, 23 were anaplastic astrocytomas (14 were *IDH*-wildtype), and one was ganglioglioma. Excluding the single ganglioglioma case present in our cohort, *IDH*-wildtype anaplastic astrocytomas in patients under 60 years old showed *RAS* mutation the most frequently (14.3%). Genetically, no other major driver mutations were identified in the anaplastic astrocytomas or the ganglioglioma, which had *RAS* mutations. The case of *RAS*-mutant anaplastic oligodendroglioma showed *IDH1* and *TERT* promoter mutations, which are known to be detected in almost all oligodendrogliomas [27]. Because of the small number of *RAS* mutant tumours, clarifying the genetic properties of *RAS* mutant tumours and discussing associations between *RAS* mutations and other driver genes is difficult, however, some studies reported the co-existing other genetic alterations in *RAS*-mutant gliomas. Clinically, the two cases of anaplastic astrocytoma with *RAS* mutation showed aggressive infiltration during the clinical course with high Ki-67 labelling index, but clinical outcomes did not differ from those of other *IDH*-wildtype anaplastic astrocytomas (Supplementary Fig. 1). The other two cases of anaplastic oligodendroglioma and ganglioglioma showed benign clinical courses. Some studies have reported *RAS* mutation as a prognostic factor in some non-neuroepithelial solid cancers [53, 54]. However, we could not explain the clinical significance of *RAS* mutation occurring in gliomas. The limitation of the present study was the rarity of *RAS* mutant gliomas due to the infrequency of *RAS* mutation in glioma. This was why the survival analysis was difficult in our cases, but it was also the same in another previous cohort. These issues should be addressed using larger cohorts in the future.

*KRAS* mutation has been reported to increase vascular endothelial growth factor (VEGF) expression and to promote the construction of a tumour vascular network [55]. However, the present study found no evidence of an aggressive vascular network such as widespread gadolinium enhancement or intra-tumoral arteriovenous shunt. *KRAS* G12D is reportedly associated with gliosis [56]. Another report suggested that *KRAS* signalling is essential for the maintenance of glioblastoma in mice, and inhibition of *KRAS* expression result in tumour apoptosis [57]. These facts proposed that *RAS* mutation has some effect on glioma maintenance and proliferation, and MAPK / PI3K pathways, which are activated by *RAS* mutation, have been suggested to be involved in the molecular pathogenesis of glioblastoma [5, 6]. Although the higher Ki-67 labelling index in the *RAS*-mutant gliomas had not been discussed previously, this may reflect the tumour proliferation activities. Some anti-RAS drugs are currently under development [19, 20], and these drugs are expected to make contributions to improving the prognosis of *RAS*-mutant glioma in the near future.

In the presented case series, recurrent tumours of case 1 (AO) and case 4 (ganglioglioma) showed no *RAS* mutations which were shown in their primary tumour. This fact may imply that tumour with *RAS* mutation was disappeared by treatment. Through direct comparison of the genomic landscape of gliomas at initial diagnosis and recurrence, a previous study showed that full set of mutations found in the initial tumour do not maintain in the recurrences and suggested that recurrent tumours are originate from cells derived at a very early stage of the evolution of tumours [58]. While *IDH1* and *TERT*p mutations, and 1p/19q codeletion assigned as the truncal events during tumour evolution [3], *RAS* mutations in glioma may be an additional alterations to development. About the primary tumours, Sanger sequencing revealed *TP53* mutation in one of these AAs, and methylation assay showed amplification of *PDGFRA* and loss of *CDKN2A/B* and *TP53* in the other. This fact proposed that RAS mutation have a potential to be a driver gene of glioma development, but its effect may be supportive compared with major truncal driver mutations like as *IDH* mutation, *TERT*p mutation and 1p/19q codeletion. Because *RAS* mutation could switch at glioma recurrence, the molecular analysis is thought to be essential for recurrent as well as primary tumours when anti-RAS treatment are conducted.

*KRAS* G12A and *KRAS* Q61K are present in 0.76 and 0.07% of cases in the Project Genomics Evidence Neoplasia Information Exchange (AACR GENIE) launched by the American Association for Cancer Research [59]. *KRAS* G12A has been identified in lung, colon, colorectal and rectal adenocarcinoma, and uterine endometrioid carcinoma, while *KRAS* Q61K has been found in colon, colorectal and pancreatic adenocarcinoma. *KRAS* G12A and *KRAS* Q61K are predictive biomarkers for the use of erlotinib, gefitinib, cetuximab, and panitumumab in patients [16–18, 60, 61]. Non-small cell lung carcinoma and colorectal carcinoma have the greatest number of therapies targeting *KRAS* G12A and *KRAS* Q61K or related pathways. *KRAS* E76D has not been reported in other types of cancer, and further study was needed whether if it has a role of an activating mutation. *NRAS* Q61R is present in 0.73% of AACR GENIE cases [59], and has been identified in cutaneous melanoma, melanoma, papillary thyroid cancer, poorly differentiated thyroid gland cancer, and colon adenocarcinoma [59]. *NRAS* Q61R is a predictive biomarker for the uses of cetuximab and panitumumab in patients [60, 61]. Further, for *NRAS*-mutant melanoma, binimetinib reportedly improves progression-free survival compared with dacarbazine [62].

Lower grade astrocytomas in our cohort contained a large number of *IDH*-wild type tumours. This fact partially results from high frequency of *TERT*p mutation. In our *IDH*-wild type tumours, 8 out of 18 DAs and 15 out of 32 AAs showed *TERT*p mutation. Nowadays, *IDH*-wild type astrocytomas with *TERT*p mutations are known as a group of astrocytomas with poor prognosis, and these tumours are supposed to be a different group from the group of common lower grade astrocytomas [63]. The diagnosis of lower grade astrocytoma without *IDH* mutation needs further discussion.

## Conclusions

We found 4 *RAS* mutations in various types of 242 gliomas. All cases involved younger adults. No clear association was identified between *RAS* mutations and clinical or genetic characteristics of tumours. Clarification of the effectiveness of anti-RAS treatments for gliomas requires further investigations in larger cohorts.

## Supplementary Information

**Additional file 1: Supplementary Fig. 1.** Kaplan-Meier curve for anaplastic astrocytomas without *IDH* mutation in the present study. The black line shows wild-type *RAS* and the red line shows mutant-type *RAS*. The *p* value is calculated as 0.98 by log-rank test.

**Additional file 2: Supplementary Fig. 2.** Chromatograms made by Sanger sequencing showing RAS mutations in the four tumours. Case 1) *KRAS* c.35 G > C, p.G12A (red arrow) in exon 2 of *KRAS*. Case 2) *KRAS* c.228 G > C, p.E76D (red arrow) in exon 3 of *KRAS*. Case 3) *NRAS* c.182 A > G, p.Q61R (red arrow) in exon 3 of *NRAS*. Case 4) *KRAS* c.180–181 TC > AA, p.Q61K (red arrows) in exon 3 of *KRAS*.

**Additional file 3: Supplementary Table 1.** Gene-specific primers used for PCR amplifications of the regions of interest in driver genes and quantitative methylation-specific PCR (qMSP) of MGMT promoter.

## Data Availability

The datasets generated and/or analysed during the current study are available from the corresponding author upon reasonable request.

## Abbreviations

AACR: American Association for Cancer Research
EGFR: Epidermal growth factor receptor
ERK: Extracellular signal-regulated kinase
FISH: Fluorescence in situ hybridization
GDP: Guanosine diphosphate
GTP: Guanosine triphosphate
ICE: Ifosfamide, carboplatin, and etoposide
MAPK: Mitogen-activated protein kinase
MEK: MAPK kinase
MGMT: O6-methylguanine-DNA methyltransferase
MLPA: Multiplex ligation-dependent probe amplification
MRI: Magnetic resonance imaging
PCR: Polymerase chain reaction
PI3K: Phosphoinositide 3-kinase
qMSP: Quantitative methylation-specific PCR
RAF: Rapidly accelerated fibrosarcoma
RAS: Rat sarcoma
RB1: Retinoblastoma
TCGA: The Cancer Genome Atlas
VEGF: Vascular endothelial growth factor
WHO: World Health Organization
