# Supplementary figures and images for: Infrequent RAS mutation is not associated with specific histological phenotype in gliomas
Source: BMC Cancer. 2021 Sep 15;21:1025. doi: 10.1186/s12885-021-08733-4 (PMC8442437; doi:10.1186/s12885-021-08733-4)

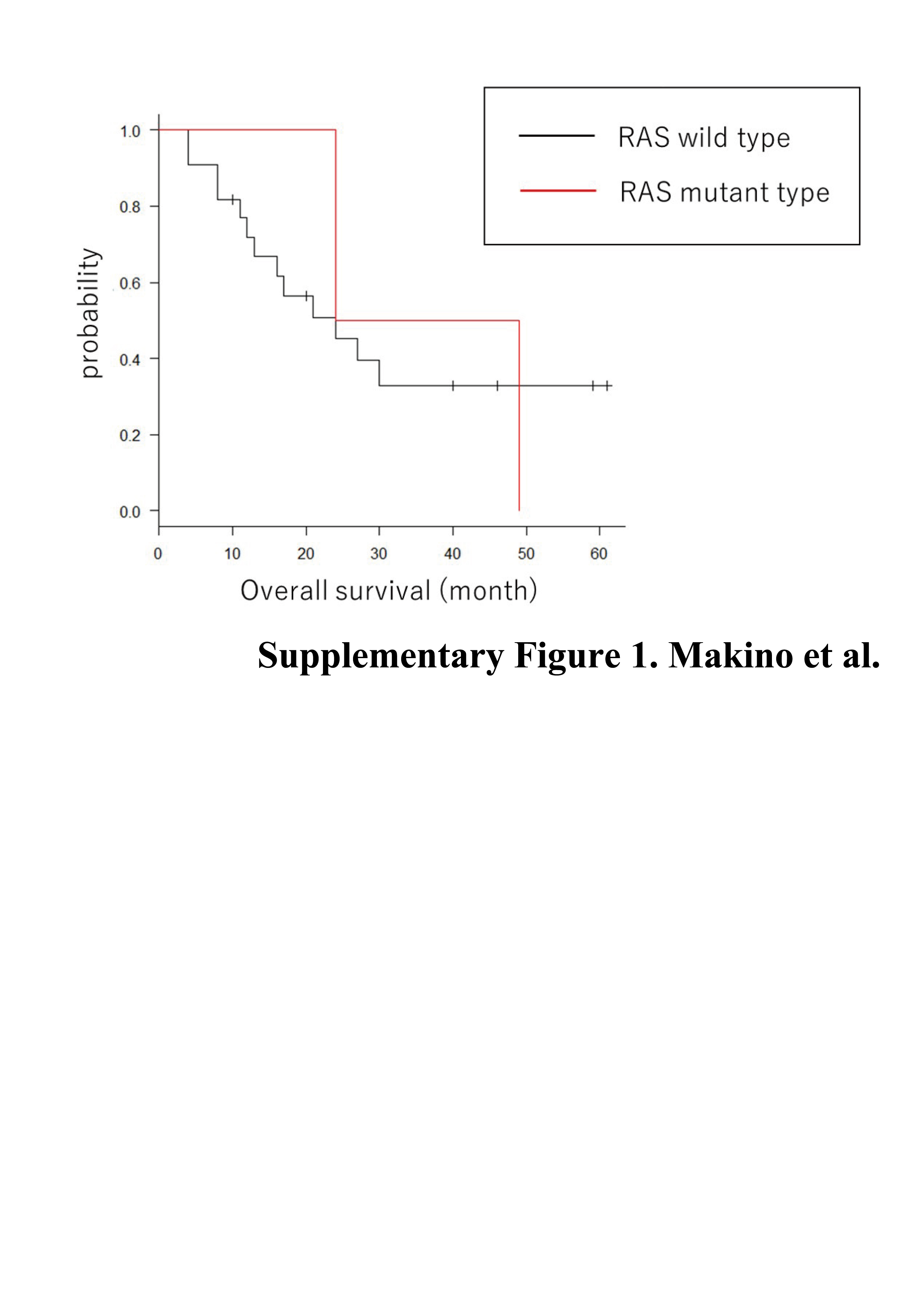

Supplement: Supplementary file 1 — Additional file 1: Supplementary Fig. 1. Kaplan-Meier curve for anaplastic astrocytomas without IDH mutation in the present study. The black line shows wild-type RAS and the red line shows mutant-type RAS. The p value is calculated as 0.98 by log-rank test. [file 12885_2021_8733_MOESM1_ESM.jpg]

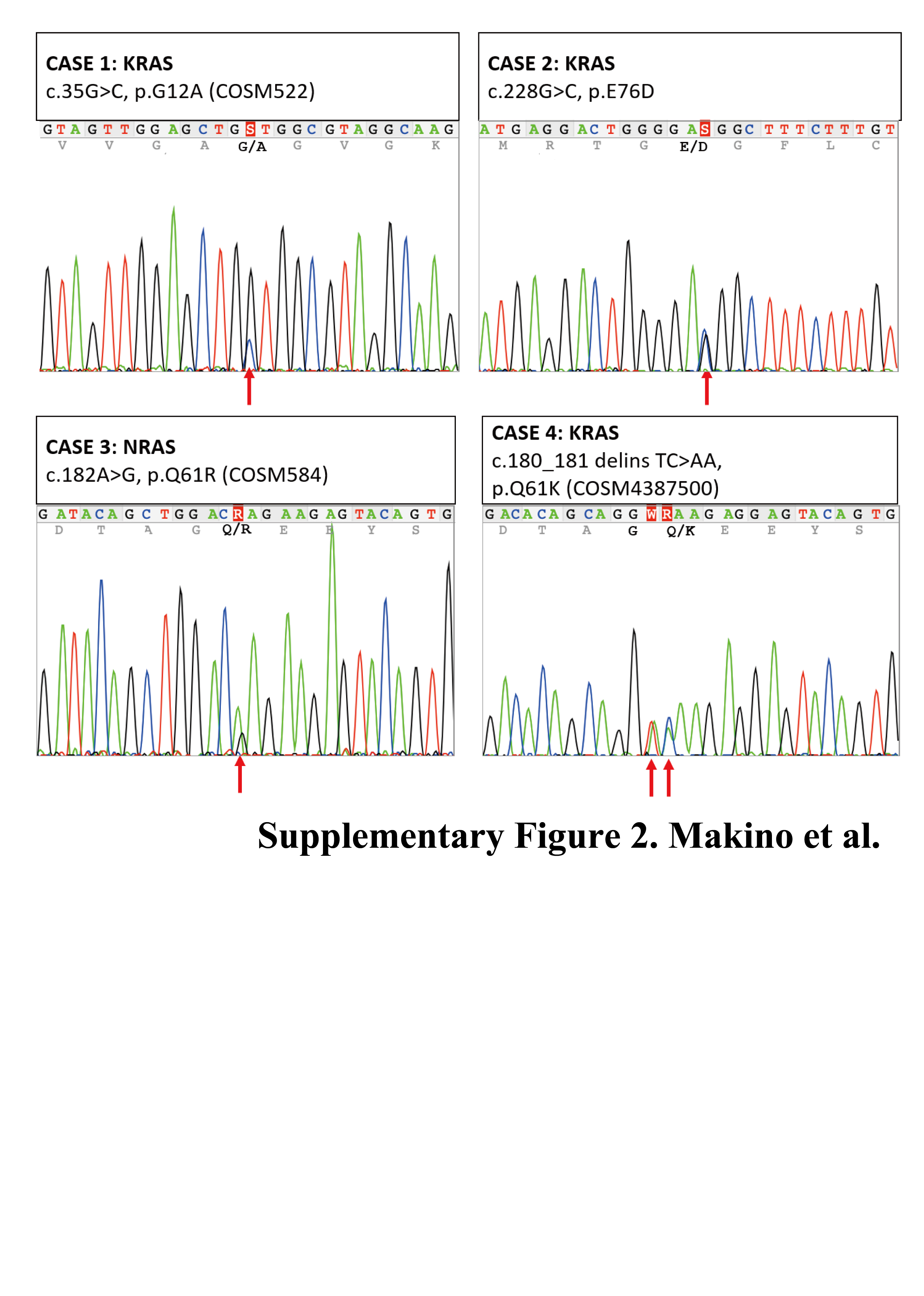

Supplement: Supplementary file 2 — Additional file 2: Supplementary Fig. 2. Chromatograms made by Sanger sequencing showing RAS mutations in the four tumours. Case 1) KRAS c.35 G > C, p.G12A (red arrow) in exon 2 of KRAS. Case 2) KRAS c.228 G > C, p.E76D (red arrow) in exon 3 of KRAS. Case 3) NRAS c.182 A > G, p.Q61R (red arrow) in exon 3 of NRAS. Case 4) KRAS c.180–181 TC > AA, p.Q61K (red arrows) in exon 3 of KRAS. [file 12885_2021_8733_MOESM2_ESM.jpg]
