# Supplementary material for: Infrequent RAS mutation is not associated with specific histological phenotype in gliomas
Source: BMC Cancer. 2021 Sep 15;21:1025. doi: 10.1186/s12885-021-08733-4 (PMC8442437; doi:10.1186/s12885-021-08733-4)
Supplement: Supplementary file 3 — Additional file 3: Supplementary Table 1. Gene-specific primers used for PCR amplifications of the regions of interest in driver genes and quantitative methylation-specific PCR (qMSP) of MGMT promoter. [file 12885_2021_8733_MOESM3_ESM.docx]

Table S1. Primer list

|  | Forward primer | Reverse primer |
| --- | --- | --- |
| IDH1 exon4 [1] | 5’- AATGAGCTCTATATGCCATCACTG-3’ | 5’- TTCATACCTTGCTTAATGGGTGT-3’ |
| IDH1 sequencing [1] | 5′-GCCATCACTGCAGTTGTAGGTTA-3′ |  |
| IDH2 exon4 [2] | 5’-TTGTTGCTTGGGGTTCAAAT-3’ | 5’-TGTGGCCTTGTACTGCAGAG-3’ |
| H3F3A exon1 | 5’-GATTTTGGGTAGACGTAATCTTCA-3’ | 5’-TACATACAAGAGAGACTTTGTCCC-3’ |
| HIST1H3B exon1 | 5’-GGGCAGGAGCCTCTCTTAAT-3’ | 5’-ACCAAGTAGGCCTCACAAGC-3’ |
| TERT [3] | 5’-TCCCTCGGGTTACCCCACAG-3’ | 5’-AAAGGAAGGGGAGGGGCTG-3’ |
| BRAF exon15 [4] | 5’-CCTAACACATTTCAAGCCCCA-3’ | 5’-CACTGATTTTTGTGAATACTGGGA-3’ |
| KRAS exon2 [5] | 5’- ACACGTCTGCAGTCAACTGG-3’ | 5’- TAACTTGAAACCCAAGGTAC-3’ |
| KRAS exon3 [5] | 5’-GCACTGTAATAATCCAGACT-3’ | 5’-CATGGCATTAGCAAAGACTC-3’ |
| HRAS exon2 [5] | 5’-AGGAGACCCTGTAGGAGGA-3’ | 5’-CTATCCTGGCTGTGTCCTG-3’ |
| HRAS exon3 [5] | 5’-AGAGGCTGGCTGTGTGAAC-3’ | 5’-GCAGCGGCATCCAGGACAT-3’ |
| NRAS exon2 [5] | 5’-GGCCGATATTAATCCGGTGT-3’ | 5’-TCCGACAAGTGAGAGACAGG-3’ |
| NRAS exon3 [5] | 5’-TTGCATTCCCTGTGGTTTTT-3’ | 5’-TGGTAACCTCATTTCCCCATA-3’ |
| MGMT qMSP-Unmet [6] | 5’- TTTGTGTTTTGATGTTTGTAGGTTTTTGT-3’ | 5’- AACTCCACACTCTTCCAAAAACAAAACA-3’ |
| MGMT qMSP-Met [6] | 5’- TTTCGACGTTCGTAGGTTTTCGC-3’ | 5’- GCACTCTTCCGAAAACGAAACG-3’ |

References

1. Bleeker FE, Lamba S, Leenstra S, Troost D, Hulsebos T, Vandertop WP, Frattini M, Molinari F, Knowles M, Cerrato A, Rodolfo M, Scarpa A, Felicioni L, Buttitta F, Malatesta S, Marchetti A, Bardelli A (2009) IDH1 mutations at residue p.R132 (IDH1(R132)) occur frequently in high-grade gliomas but not in other solid tumors. Hum Mutat 30: 7-11 doi:10.1002/humu.20937

2. Yan H, Parsons DW, Jin G, McLendon R, Rasheed BA, Yuan W, Kos I, Batinic-Haberle I, Jones S, Riggins GJ, Friedman H, Friedman A, Reardon D, Herndon J, Kinzler KW, Velculescu VE, Vogelstein B, Bigner DD (2009) IDH1 and IDH2 mutations in gliomas. N Engl J Med 360: 765-773 doi:10.1056/NEJMoa0808710

3. Arita H, Narita Y, Fukushima S, Tateishi K, Matsushita Y, Yoshida A, Miyakita Y, Ohno M, Collins VP, Kawahara N, Shibui S, Ichimura K (2013) Upregulating mutations in the TERT promoter commonly occur in adult malignant gliomas and are strongly associated with total 1p19q loss. Acta Neuropathol 126: 267-276 doi:10.1007/s00401-013-1141-6

4. Arita H, Yamasaki K, Matsushita Y, Nakamura T, Shimokawa A, Takami H, Tanaka S, Mukasa A, Shirahata M, Shimizu S, Suzuki K, Saito K, Kobayashi K, Higuchi F, Uzuka T, Otani R, Tamura K, Sumita K, Ohno M, Miyakita Y, Kagawa N, Hashimoto N, Hatae R, Yoshimoto K, Shinojima N, Nakamura H, Kanemura Y, Okita Y, Kinoshita M, Ishibashi K, Shofuda T, Kodama Y, Mori K, Tomogane Y, Fukai J, Fujita K, Terakawa Y, Tsuyuguchi N, Moriuchi S, Nonaka M, Suzuki H, Shibuya M, Maehara T, Saito N, Nagane M, Kawahara N, Ueki K, Yoshimine T, Miyaoka E, Nishikawa R, Komori T, Narita Y, Ichimura K (2016) A combination of TERT promoter mutation and MGMT methylation status predicts clinically relevant subgroups of newly diagnosed glioblastomas. Acta Neuropathol Commun 4: 79 doi:10.1186/s40478-016-0351-2

5. Chang YS, Yeh KT, Hsu NC, Lin SH, Chang TJ, Chang JG (2010) Detection of N-, H-, and KRAS codons 12, 13, and 61 mutations with universal RAS primer multiplex PCR and N-, H-, and KRAS-specific primer extension. Clin Biochem 43: 296-301 doi:10.1016/j.clinbiochem.2009.10.007

6. Esteller M, Hamilton SR, Burger PC, Baylin SB, Herman JG (1999) Inactivation of the DNA repair gene O6-methylguanine-DNA methyltransferase by promoter hypermethylation is a common event in primary human neoplasia. Cancer Research 59: 793-797
